# Implementation of National Health Informatization in China: Survey About the Status Quo

**DOI:** 10.2196/12238

**Published:** 2019-02-21

**Authors:** Chen Li, Xiangdong Xu, Guanghua Zhou, Kai He, Tianliang Qi, Wei Zhang, Feng Tian, Qinghua Zheng, Jianping Hu

**Affiliations:** 1 School of Electronic and Information Engineering Xi'an Jiaotong University Xi'an China; 2 Shanxi Province Key Laboratory of Satellite and Terrestrial Network Technology Research and Development Xi'an Jiaotong University Xi'an China; 3 Department of Information Technology Center of Statistics and Health Informatics National Health Commission of People’s Republic of China Beijing China; 4 College of Medicine and Health Management Tongji Medical College Huazhong University of Science and Technology Wuhan China

**Keywords:** health policy, health informatization, electronic health record

## Abstract

**Background:**

The National Health and Family Planning Commission (NHFPC) in China organized a nationwide survey to investigate the informatization in hospitals and regional Health and Family Planning Commissions (HFPCs) in 2017. The survey obtained valid results from 79.69% (2021/2536) of major hospitals and 81% (26/32) of provincial and 73.1% (307/420) of municipal HFPCs. The investigated topics covered hardware infrastructure, information resources, applications, systems, and organizations in health informatics.

**Objective:**

This study aimed to provide evidence collected from the survey regarding China’s health informatization and assist policy making regarding health informatics in the 13th Five-Year Plan of China.

**Methods:**

Based on the survey, the paper presented the status quo of China’s health informatization and analyzed the progress and potential problems in terms of the country’s health information development policies.

**Results:**

Related policies have helped to construct 4-level information platforms and start converging the regional data to the 3 centralized databases. The principle of informatics has been transiting from finance-centered to people-centered. Alternatively, the quality, usability, and interoperability of the data still need to be improved.

**Conclusions:**

The nationwide survey shows that China’s health informatization is rapidly developing. Current information platforms and databases technically support data exchanging between all provinces and cities. As China is continuing to improve the infrastructure, more advanced applications are being developed upon it.

## Introduction

**Figure 1:**
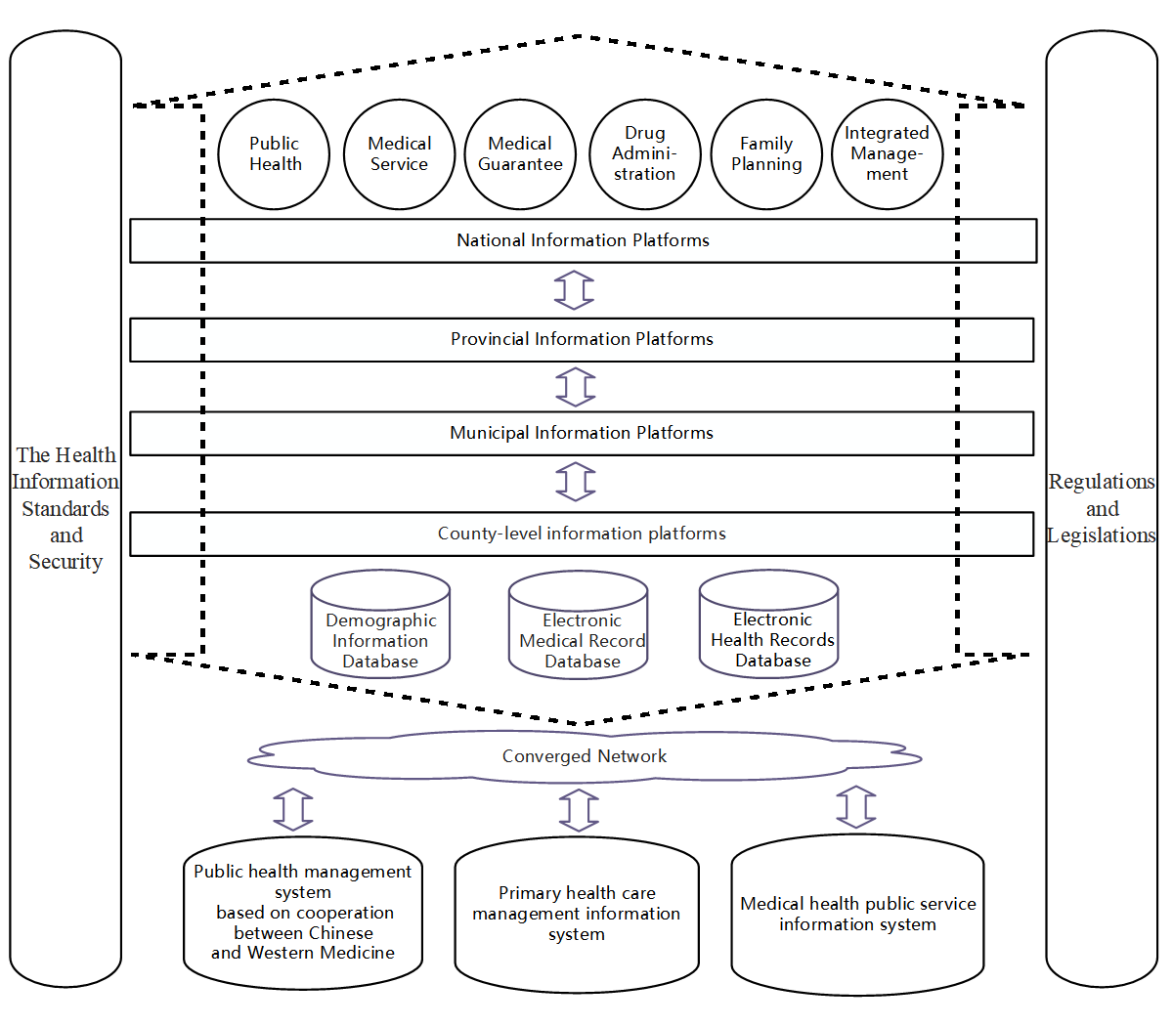
The 4631-2 Project framework of health informatization.

In May 2011, the 12th Five-Year Plan and its corresponding subplans made by the State Council of the People's Republic of China (PR China) were approved. One of the subplans, Instructions for Accelerating Population Health Informatization [1], emphasized that health informatization was a crucial part of the national informatization framework and an important aspect of continuing reform of medical health in China. The difficulty of making a comprehensive plan of health informatization for a country with 1.4 billion people was probably beyond imagination. In China, the National Health and Family Planning Commission was responsible for proposing the plan and the related policies. In 2010, the NHFPC initialized the 3521 Project, which was known as China's health informatization road map and later upgraded to the 4631-2 Project in 2013 [1,2]. Figure 1 illustrates the planned 4631-2 Project and its numeric representations:

- The 4 stands for the 4-level information platforms, which include the national information platforms, provincial information platforms, municipal information platforms, and county-level information platforms. On each platform, the medical data from different regions are integrated and shared.
- The 6 stands for 6 primary types of applications, including public health, medical service, medical guarantee, drug administration, family plan, and integrated management. The 6 types of applications are deployed on each of the 4-level information platforms.
- The 3 stands for 3 demographic health information databases (DHID) constructed on each of the 4-level information platforms, including a demographic information database (DID), electronic medical record database (EMRD), and electronic health record database (EHRD) [3]. The DID contains fundamental population information, family planning service management information, and floating population management information, etc. The EMRD stores all information from the electronic medical record. The EHRD regards the residents’ personal health information as the key and stores all information from the electronic health record (EHR), which is defined as “digitally stored health care information about an individual’s lifetime with the purpose of supporting continuity of care, education, and research and ensuring confidentiality at all times” [4].
- The 1 stands for one efficient and centralized network covering all kinds of health care institutions at all levels (including Chinese medical institutions).
- The 2 stands for health information standards, security, regulations, and legislation.

In October 2016, the State Council of PR China issued the Plan for Healthy China, in which the importance of informatization in health care was reemphasized [5]. Subsequently, the NHFPC released the National Population Health Informatization Development Plan (NPHIDP13) in January 2017 containing the fundamental policies [6]. At the beginning of the NPHIDP13, NHFPC conducted a nationwide survey requiring the participation of thousands of hospitals and all provincial and municipal health and family planning commissions (HFPCs). Based on the survey and its analysis, the paper discussed health informatization progress in China and its challenges. It showed that although the country’s health informatization had been initialized relatively later than many countries, it has been developing at a rapid and steady pace. The initial goal of constructing the EHR systems and sharing data among regions had been achieved. With the construction of the EHRD, the development of health informatization had started transiting from finance-centered to people-centered. Some prominent problems remain: low data quality, data usability, and interoperability are yet to be solved, and the national structure of 4-level information platforms has been really challenging. The survey investigated the progress of health informatization and would support policy- and strategy-making. To our knowledge, this paper is the first on research about health informatization based on a nationwide survey in China, and similar research is rare.

## Methods

The survey consists of 3 comprehensive questionnaires including the Hospital Informatization Questionnaire, the Regional HFPC Informatization Questionnaire, and the Big Data Technology in Health Informatization Questionnaire. Each questionnaire was developed by a group of 8 experts and covers a range of key aspects of informatization, including administration, applications, hardware infrastructure, big data applications, information exchange platforms, information security, and related standards. In all, 32 provincial HFPCs, 420 municipal HFPCs, and 2536 major hospitals were invited. Valid responses came from 81% (26/32) of provincial HFPCs, 73.1% (307/420) of municipal HFPCs, and 79.69% (2021/2536) of hospitals.

The NHFPC is the state administrative department for health-related affairs in the State Council of PR China. The provincial and municipal HFPCs are local district branches under the supervision of the NHFPC. A provincial HFPC was responsible for the health affairs of the corresponding province, including ensuring public health; implementing health policies; promoting health care reform; and managing affiliated medical administrations, hospitals, and schools. Similarly, the municipal HFPC has the same responsibility at the municipal level. Among 2021 hospital respondents, there were 42 NHFPC-affiliated hospitals, 371 provincial NHFPC-affiliated hospitals, and 1608 municipal NHFPC-affiliated hospitals.

Similar research regarding health informatization has been carried out in a number of countries [7-10]. Compared with this research, our information was collected from an official survey conducted by the country’s health ministry (NHFPC), and the survey covered a wider range about health informatization, including hardware and software of health infrastructure, national data exchanging platforms, health informatization applications, investment, and staffing.

## Results

### Three Phases of Informatization

The 4631-2 Project, the guidance of health informatization in China, consisted of 3 phases. The first phase was to improve health information technology (IT) hardware infrastructure and the application systems of routine businesses and set up the information standards and security systems. The second phase was to construct the information platform levels according to region levels. The hierarchy and mechanism enabled data exchange between different regions and applications. The third phase was to apply big data technologies, artificial intelligence (AI), and Internet Plus (China’s plan to manage its information superhighway) proposed in a government work report from March 5, 2015, that refers to the application of the internet and other IT in conventional industries [11] and enables advanced applications (eg, predictive modeling, clinical decision support, disease or safety surveillance, public health, and research) [2,12].

### People-Centered Health Information Infrastructure

The various medical information systems, such as EHR systems, were the fundamental data resources. The continuously improved EHR systems and 3 DHIDs converged the data and gradually became the central resources. The NHFPC had promoted nationwide deployment of EHR systems and regular data synchronization into the EHRD to compile a complete EHR for each citizen regardless of where the original data were generated. Each region’s local HFPC was responsible for the implementation of the EHRD. The survey showed that 86% (18/22) of provincial HFPCs and 75.6% (232/307) of municipal HFPCs had established the EHRD as of March 2017. Meanwhile, 71% (15/21) of provincial HFPCs and 70.4% (216/307) of municipal HFPCs had EHRs for 70% population in the corresponding regions. In addition, 16.19% (3,880,879/23,956,343) of provincial and 14.31% (271,920/1,900,209) of municipal HFPC EHRs were built in 2016 alone.

Data from 6 types of applications and related systems were organized into the other 2 core databases, the DID and EMRD. The survey showed that 96% (22/23) of provincial HFPCs had built DIDs and 48% (10/21) of provincial and 31.6% (97/307) of municipal HFPCs had started constructing EMRDs. The NPHIDP13 had explicitly required the data from 6 applications and other related systems to be available to 3 DHIDs. It had also required that the 3 DHIDs should be integrated following the people-centered principle. Meanwhile, NPHIDP13 had explicit criteria about system consistency, accuracy, and integrity.

### Data Exchanging on the Four-Level Information Platforms

Centralized medical data had significant potential for applications and research. Enabling information sharing within and across regions had been attempted by a few countries and was demonstrated to be difficult for various reasons [13-17]. The 4631-2 Project supported regional informatization that collected data from 6 primary types of applications, 3 population health information databases (PHIDs), and other public health systems. Systematic informatization also promoted sharing data within and across regions. As the development of 4-level information platforms was the core task of regional informatization, the platforms’ construction had been one of the key tasks in the Instructions for Accelerating Population Health Informatization [1]. In the 4-level information platforms, most data were generated at the provincial and municipal platforms, so these 2 levels of the platforms naturally became the focuses of the survey. As of March 2017, 32% (7/22) of provincial and 35.2% (108/307) of municipal HFPCs had built the information platforms. All provincial and all but 17 (6%) of the municipal HFPCs had started establishing information platforms. More importantly, all 32 provincial platforms had been connected with the national platforms.

The 4-level information platforms offered 2 advantages. The first was centralization of storing and sharing health information. The 4-level information platforms also connected public health information systems with medical and health service institutions within regions. The integration and sharing within the information platforms would directly improve the management efficiency of health administrations, for example, by providing panoramic information of the entire country during an epidemic breakout. The second was to unify management for EHRs. Based on the information platforms, unified management could facilitate various nationwide standardization.

### Advanced Applications

Advanced applications based on the converged data and technologies like big data, AI, and Internet Plus were considered to have great potential to benefit the people and the health care industry [18-20]. The NPHIDP13 plan had been designed with this consideration. The survey showed that 67% (16/24) of provincial and 31.8% (100/314) of municipal HFPCs and 32.35% (647/2000) of hospitals had implemented various big data applications. Business intelligence reports, medical quality control warnings, clinical medical records, and related data retrieval had been well received by the users. A poll within the survey showed that clinical quality control and chronic disease management based on big data were most appreciated by medical professionals. At the same time, the poll showed that 1180 (59.00%), 1062 (53.10%), and 944 (47.20%) of the 2000 hospitals expected more funds, consistent standards, and interinstitution cooperation, respectively, regarding big data research and application development.

The combination of big data, AI, and Internet Plus still needed to overcome technical and technological challenges to reveal the true potential and support the most demanded applications (eg, precision medicine and AI-assisted clinical decision support systems) [21-23]. One of the issues was the collection and processing of multimodel data from various sources. The NPHIDP13 had tried to structure the data as much as possible. The Big Data Technology in Health Informatization Questionnaire showed that 45.10% (902/2000) of hospitals had structured health big data, 41.40% (828/2000) had semistructured electronic medical record health big data, 28.50% (570/2000) had unstructured medical image data, and 6.00% (120/2000) had unstructured omics data. For regional HFPCs, 24% (5/21) of provincial and 15.9% (50/314) of municipal HFPCs had unstructured medical image data, and 48% (10/21) of provincial and 1.9% (6/314) of municipal HFPCs had unstructured omics data. Meanwhile, the unstructured data, which could be 80% or 85% of overall data, needed more professional curating staff and technologies like natural language processing and image processing to automatically process it [24,25].

### Investment and Staffing

The national health informatization project was extremely complex and expensive, but the Chinese government played an essential and supportive role in the project: increases were not seen in local medical service prices and data privacy and usability were better assured. As of March 2017, the 32 provincial platforms received ¥1.87 billion (US $276.6 million) from the central government (not including funds from local governments). During the 12th Five-Year Plan, the central government invested more than ¥10 billion (US $1.5 billion) into health informatization [1,3,6].

Since health care reform in 2009, the number of doctors (approximately 1,730,000 in 2015) has increased by 3.3% annually. The annual rate of new nurses is even larger (9.9%, approximately 472,000 in 2015). The increasing rate of pharmacists is 3.1% (approximately 109,000 in 2015) [26-28]. In comparison with the increasing number of medical professionals, the number of IT professionals is relatively low. Often, the responsibility of the IT departments must be limited in coordinating the needs with the health IT service providers due to a limited number of IT staff. According to the survey, 19% (4/21) of provincial and 85.0% (267/314) of municipal HFPCs had fewer than 9 IT staff members. For the hospitals, only 7.00% (140/2000) had more than 20 full-time IT staff members; the number of IT staff was not enough to meet the actual work needs. The survey showed that 76.00% (1520/2000) of medical institutions expressed the lack of IT staff and the difficulty of recruitment.

### Standards and Certification

**Figure 2:**
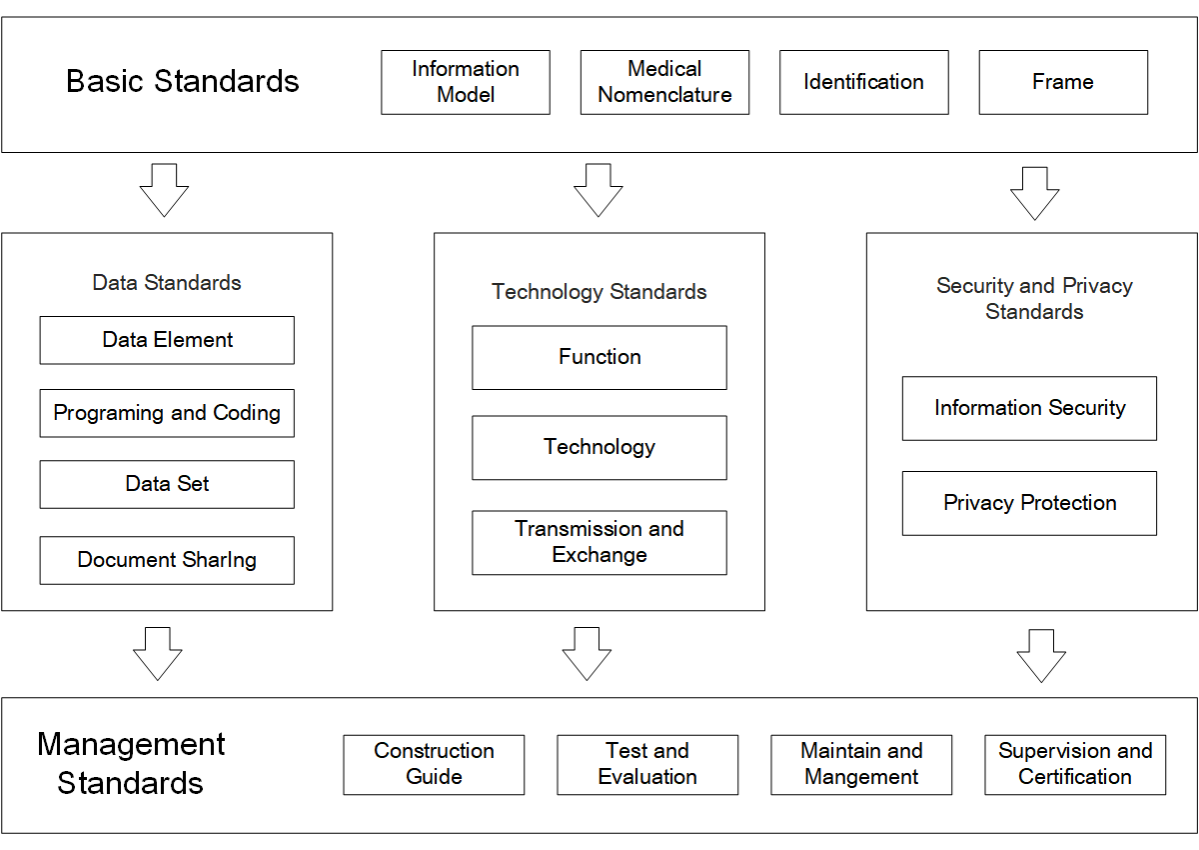
Framework for information standardization.

Standards, motivation, and credibility were 3 keys to effective interoperability of health information technology [29]. Standards provided the possibility of efficient communications, motivation promoted the usability and consequently created more data, and credibility ensured a trustworthy environment for using data. China’s information standardization framework comprises 3 parts: data standards, technology standards, and security and privacy standards (Figure 2). Since 2010, 283 national health informatization standardization projects had been approved and initiated, ranging from data acquisition and exchange to information management, storage, cataloging, and security, etc. Among the approved standards, 33 have been adopted by consortiums. Of 250 planned industry standards, 209 have been published, 16 will be published soon, another 16 have passed preliminary assessment, and 10 are under development.

Informatization standards not only cover hardware-related issues such as data acquisition, storage, and transmission, called hard standards, but also software-related issues such as metadata standards, information usage standards, supervision and certification, called soft standards. Generally, soft standards are relatively immature compared with hard standards. Furthermore, hard and soft standards should be better integrated within the information standardization framework (Figure 2). Many existing information standards still play separate roles, forming the biggest barriers for interoperability. With awareness of these issues, the NHFPC is making efforts to improve the synergy of health informatization standards to enhance interoperability.

**Figure 3:**
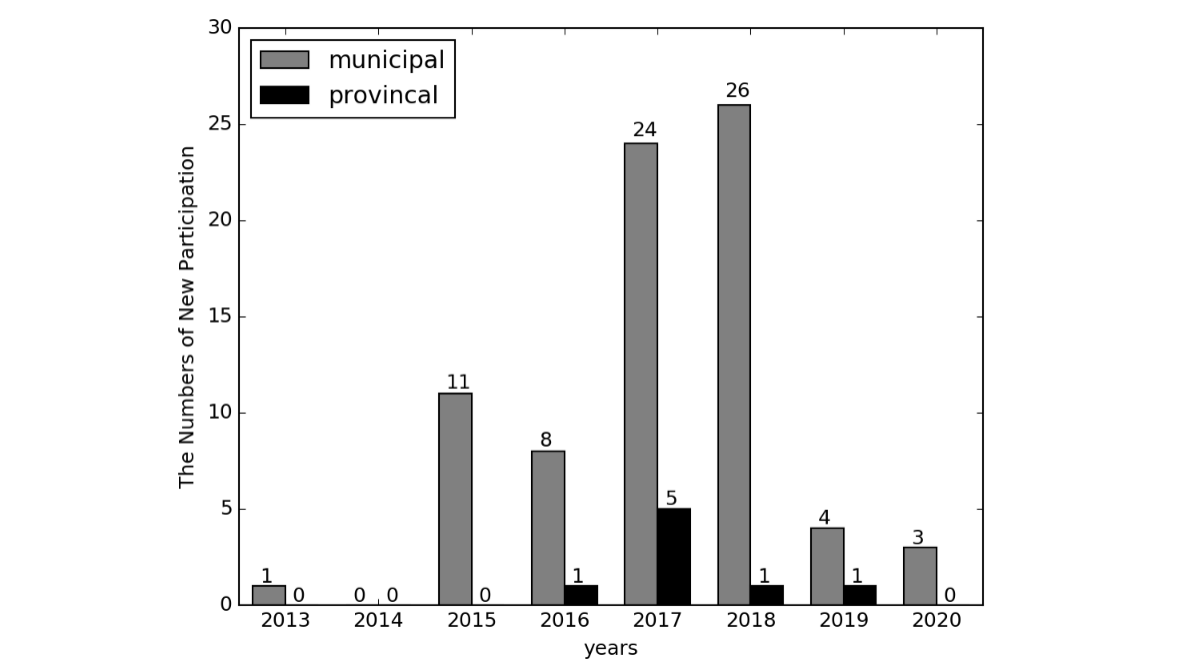
Schedule of regional Health and Family Planning Commissions obtaining certifications.

To help the regional HFPCs and hospitals stay informed about standardization and better implementation of standards, a series of certification programs has been launched by the NHFPC. The National Medical Health Information System’s Interconnection and Interoperability Standardization Certification was started in 2013. The certification covers a wide range of data standards, document sharing, platform functions, hardware infrastructure, information security, business applications, etc [30]. As shown by the survey, 9.1% (24/263) of municipal platforms participated in the certification in 2017. Because the program for provincial platforms just started, only one provincial platform has participated. Meanwhile, 46% (8/17) of provincial and 31.3% (77/246) of municipal HFPCs have a clear timeline for taking part in the program. Figure 3 shows the regional HFPCs’ planned schedule of participating the program.

## Discussion

### Principal Findings

Compared with 59.9% of eligible nonprofit hospitals that get funding in the United States [31], almost all major hospitals in China are public and nonprofit. The Chinese government is responsible for funding support and therefore plays a more active role in health informatization.

One of the key achievements in recent years was the development of nationwide 4-level information platforms. It was an ambitious and challenging task that established a certain level of connectivity between HFPCs in the country. This might enhance information sharing and collaboration and may directly support allocating health care resources according to regional differences. For medical professionals, it largely improved information accessibility—for example, to be able to make rapid diagnoses based on more complete information or reduce the number of reexamination cases. For patients, it improved the accessibility of health information and enabled interregional medical health and insurance services. For health administrations, it delivered real-time intelligence of dynamic situations about health service resources to support scientific management, such as inspecting and maintaining reasonable medical expenses. The potential of converged data was still far from being fully exploited and many more data applications were still under development. The survey showed that 71% of provincial HFPCs regarded developing and improving applications as a main task for the future.

Another more serious problem is low data quality. According the analysis of the survey, empty or invalid data, disorders of disease description language, lack of retrieval tools, lack of metadata management, and inability to handle the text data of electronic medical records are prominent problems in China’s health informatization. Only 66.8% of municipal NFPCs implements or plans to implement data quality management system standards. The problem is serious and may cause unexpected barriers for the usability of the 3 databases and the platform. Without high-quality local data, centralized medical data on the 4-level information platforms won’t play a real role and advanced applications based on converged data and technologies like big data and AI will not have a solid foundation. The survey also reported that 74% of provincial HFPCs put this at the top of the list of problems to be solved. To summarize, we list the best practices and corresponding barriers and successes of the national health informatization implementation in Table 1.

**Table 1:** Best practices and corresponding main barriers and successes in the implementation of national health informatization.

Best practices	Main barriers	Successes
Three demographic health information databases	Low data quality	Large enough coverage for population
Four-level information platforms	Difficultly exchanging data	National health data exchanging
Advanced informationalized applications	Unstructured data processing	Big data, AI, and Internet Plus applications
Established standards	Lack of synergy of standards	A total of 283 national standardizations were approved

### Conclusions

Health informatization in China started in the relative recent years and has developed at a very rapid pace. Its objective is to enable advanced clinical research and applications at the beginning while the IT infrastructure is built. Initially, informatization aimed to improve efficiency and administration of finances. Gradually, informatization transited from finance-centered to people-centered in order to bring full potential in terms of health outcomes. The transition was naturally occurring while the goal of informatization was explicitly set to bring more benefits for every patient in NPHIDP13.

According to NPHIDP13, personalized health care services and products based on perception technology will start being applied and promoted by 2020; DHIDs storing individual lifelong records for the entire population will be eventually built; and novel technologies, which might bring revolutionary outcomes in health care and research, were considered while designing the current systems and planning future advanced applications. Generally, NPHIDP13 planned the projects for the period of 2016 to 2020 to solve remaining problems. They include upgrading current systems with new technologies, continuing development of applications of key businesses, improving coverage of the population in 3 DHIDs, and improving interconnection and interoperability between the 4-level platforms.

## Abbreviations

AI: artificial intelligence
DHID: demographic health information database
DID: demographic information database
EHR: electronic health record
HFPC: Health and Family Planning Commission
EHRD: electronic health record database
EMRD: electronic medical record database
IT: information technology
NHFPC: National Health and Family Planning Commission
NPHIDP13: National Population Health Informatization Development Plan
PHID: population health information database
PR China: People’s Republic of China
